# Pupil responses to colorfulness are selectively reduced in healthy older adults

**DOI:** 10.1038/s41598-023-48513-7

**Published:** 2023-12-13

**Authors:** Janneke E. P. van Leeuwen, Amy McDougall, Dimitris Mylonas, Aida Suárez-González, Sebastian J. Crutch, Jason D. Warren

**Affiliations:** 1 Dementia Research Centre, UCL Queen Square Institute of Neurology, University College London, 8-11 Queen Square, London, WC1N 3AR UK; 2 The Thinking Eye, ACAVA Limehouse Arts Foundation, London, UK; 3 Department of Medical Statistics, London School of Hygiene & Tropical Medicine, London, UK; 4 Faculty of Philosophy, Northeastern University London, London, UK

**Keywords:** Ageing, Colour vision, Autonomic nervous system, Neural ageing

## Abstract

The alignment between visual pathway signaling and pupil dynamics offers a promising non-invasive method to further illuminate the mechanisms of human color perception. However, only limited research has been done in this area and the effects of healthy aging on pupil responses to the different color components have not been studied yet. Here we aim to address this by modelling the effects of color lightness and chroma (colorfulness) on pupil responses in young and older adults, in a closely controlled passive viewing experiment with 26 broad-spectrum digital color fields. We show that pupil responses to color lightness and chroma are independent from each other in both young and older adults. Pupil responses to color lightness levels are unaffected by healthy aging, when correcting for smaller baseline pupil sizes in older adults. Older adults exhibit weaker pupil responses to chroma increases, predominantly along the Green–Magenta axis, while relatively sparing the Blue–Yellow axis. Our findings complement behavioral studies in providing physiological evidence that colors fade with age, with implications for color-based applications and interventions both in healthy aging and later-life neurodegenerative disorders.

## Introduction

Colors are neither stable nor uniform sensations, due to natural variations in the retinal cone photoreceptors^1–3^ and complex interactions between environmental factors, neural processing dynamics, and psychosocial influences^4–11^. Due to this complexity, the relationships between material aspects of colors and how they are perceived by human beings are notoriously difficult to study and still not fully understood. It is known that healthy aging also affects color perception, but the underlying mechanisms are not well characterized yet. One of the aspects of color perception that has been found to decline with older age, is the perception of colorfulness. Behavioral research has shown that older adults judge surface colors as less saturated (colorful) than young adults^12,13^. How this process is grounded in the visual system is not clear however. A study which measured the visual evoked potentials (VEP) responses to pure luminance contrast (with constant color) and pure color contrast (Red–Green equiluminant gratings) found that the visual cortex showed a uniform decline in VEP responses to both luminance and color contrast in older adults^14^. However, another study which combined VEP with behavioral measurements found that chromatic processing was significantly affected by age, whereas achromatic processing was not^15^. Color discrimination has also been shown to decline with increasing older age, but studies have reported differing results with speculations about the possible loci of change ranging from low-level retinal processing to top-down cortical influences. Some studies have found that older adults struggle in particular to discriminate between colors on the green/blue spectrum^16,17^. It has been proposed that this might be attributed to a reduced retinal transmission of short wavelengths (blue light), due to the yellowing of the crystalline lens in older age^17,18^. A reduced retinal transmission of short wavelengths in older age has also been proposed as an explanatory mechanism for the observation that older adults selected a green hue that was closer to the blue spectrum than the choice of young adults, when asked to select a color patch closest to pure, or ‘Unique Green’^13,18,19^. This seems counter-intuitive, but it has been suggested that this observed perceptual amplification of blue in older adults might be the effect of cortical compensation mechanisms which ensure that our perception of colors remains relatively constant across the life span despite changes in lower-level color processing due to aging^13,18–22^. However, to our knowledge no neural correlates of these assumed compensatory cortical mechanisms have been found to date. Furthermore, not all the available psychophysical literature supports the proposed reduced sensitivity to blue light in older adults. Some studies have reported a more evenly distributed decline in color discrimination across the visible spectrum in older adults, which has been speculatively attributed to a reduced retinal illuminance due to a global decline in photoreceptors and retinal ganglion cells^20,23^. In addition to these color discrimination studies, which relied on explicit color perception, pupillometry studies have also cast doubt on the premise that older adults are less sensitive to blue light. Daneault et al.^24^ found no difference in pupil responses to green or blue light in older adults when they controlled for the smaller baseline pupil sizes in older adults, and Rukmini et al.^25^ showed that the yellowing of the crystalline lens did not selectively reduce pupil responses to blue light, compared to red light.

As illustrated above, pupillometry can be a useful instrument to further illuminate the effects of aging on the dynamics of color processing, but only limited research has been done in this area so far. Pupil responses have been shown to correspond with both bottom-up and top-down visual information signaling between the retina and the visual cortex, as well as higher cortical systems^26–31^. The Pupillary Light Reflex, which causes the pupils to constrict in response to light, is regulated by the Edinger-Westphal (EW) nucleus in the midbrain. The EW nucleus is part of the oculomotor nuclear complex (ONC) and receives input from all types of retinal photoreceptors, including rods which process only achromatic information and cones which process both chromatic and achromatic information^29,31–36^. Achromatic luminance information—thought to code for dynamic visuospatial properties—is generated by many types of retinal photoreceptors and is processed mainly in the magnocellular layer of the lateral geniculate gyrus (LGN). Chromatic information from the retinal long (L) and medium (M) wavelength sensitive cones (commonly referred to as the Red—Green channel) is processed in the parvocellular layer of the LGN, but achromatic sensations caused by L and M cone stimulation have also been shown to activate the parvocellular layer^33,35^. The koniocellular layer in the LGN (commonly referred to as the Blue—Yellow channel) processes chromatic information from the short (S) wavelength sensitive retinal cones in relation to the combined M and L cone input^37–41^. The different chromatic and luminance aspects of color vision that are processed in the magno-, parvo-, and koniocellular layers of the LGN are conveyed in separate neural pathways to the visual cortex. These separate neural pathways for color and luminance information suggests there might be a certain level of independence between luminance and color processing, but no behavior or neural pathway has yet been identified that is solely informed by color, completely independent of luminance^42,43^.

In summary, further research is required to illuminate how the different components of color information are processed by the human visual system, and how this is affected by aging. The close alignment between visual pathway signaling and pupil dynamics offers a promising non-invasive method to investigate this and previous research has shown that pupil responses are sensitive to changes in the wavelength (hue) purity, intensity, contrast, and duration of a light stimulus^44–47^. The effects of healthy aging on pupil responses to different color components have not been studied yet however. Advancing our knowledge on this topic will improve our understanding of the neurobiology of color perception, and has practical implications for everyday color use and applications designed for older adults. It will furthermore enrich our understanding of later-life neurodegenerative diseases (e.g., Alzheimer’s disease, posterior cortical atrophy (PCA), and Parkinson’s disease), which often affect the visual system as well^48–50^. In this study we aimed to address this by modelling the effects of color lightness and chroma (colorfulness) on pupil responses in young and older adults, in a closely controlled passive viewing experiment with 26 broad-spectrum digital color fields.

Based on an analysis of the existing literature, we hypothesized that (i) there will be a level of independence between the effects of color lightness and chroma on pupil responses in both young and older adults, (ii) pupil responses to increasing levels of chroma (colorfulness) are reduced in older adults, but pupil responses to color lightness levels are unaffected by healthy ageing when correcting for smaller baseline pupil sizes.

Expanding on the second hypothesis, we also explored whether intensity levels of specific hues might be driving the pupillary response to chroma. For this purpose we analyzed the pupil effects of the relative saturation levels of Green, Magenta, Yellow, and Blue as defined in the CIELAB perceptual color space by the a* coordinate (Green opposite Magenta) and the b* coordinate (Yellow opposite Blue).

## Results

### Color pupillometry experiment

**Figure 1:**
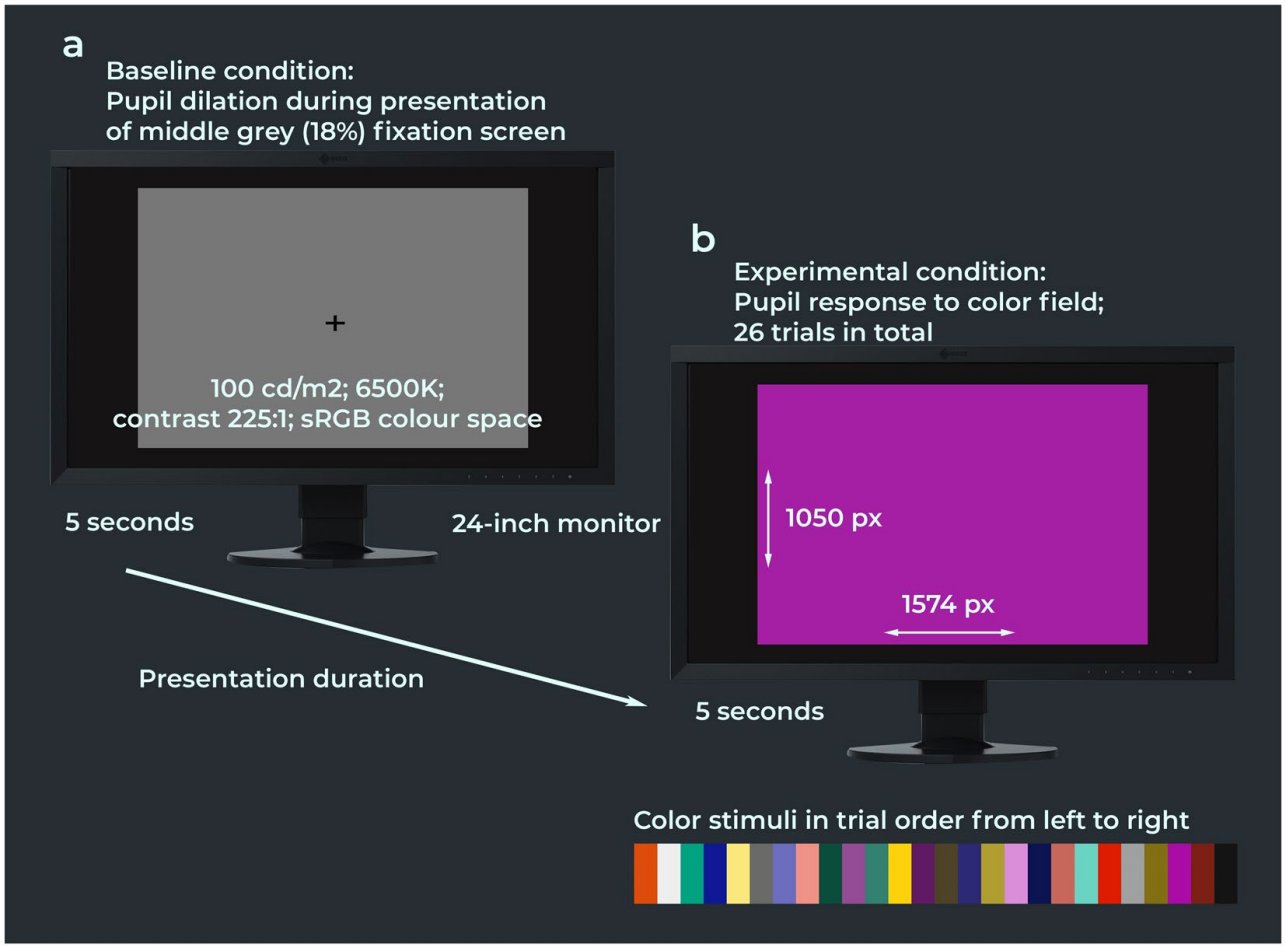
Procedure of the color pupillometry experiment. The experimental stimuli consisted of 26 broad-spectrum color fields which were shown in a black-out room on an Eizo ColorEdge CG2420 24-inch LCD monitor in pseudo-randomized to prevent that variations of the same color category were shown in direct succession (e.g., light blue and dark blue). There were 26 trials in total. Each trial consisted of a 5-s baseline condition (**a**) and an experimental condition (**b**) during which a color field was shown for 5 s. Pupil diameters were measured continuously at a frequency of 1000 Hz with an SR Research Eyelink 1000 Plus table-mounted eye-tracking camera. Further information on the experimental design and color selection can be found under “Materials and methods” and in the Supplementary Materials.

Seventeen healthy young adults (F = 10, mean age = 27.7, SD = 2.5) and twenty healthy older adults (F = 10, mean age = 64.4, SD = 8.3) were recruited for this study. The color pupillometry experiment took place in a black-out room where participants were shown 26 digital broad-spectrum color fields (1574 × 1050 px) against a black background on an Eizo ColorEdge CG2420 24-inch LCD monitor which was calibrated in the sRGB color space. Each trial started with the presentation of a mid-grey (18%) fixation screen (1574 × 1050 px) against a black background for 5 s, after which the color stimulus was presented for 5 s. Pupil diameters were measured continuously at a frequency of 1000 Hz with an SR Research EyeLink 1000 Plus table-mounted eye-tracking camera. Participants could recompose at their own pace between each color trial. Figure 1 illustrates the experiment procedure. Further information on the experimental design can be found under “Materials and methods” and in the Supplementary Materials.

### Statistical analysis methods

**Figure 2:**
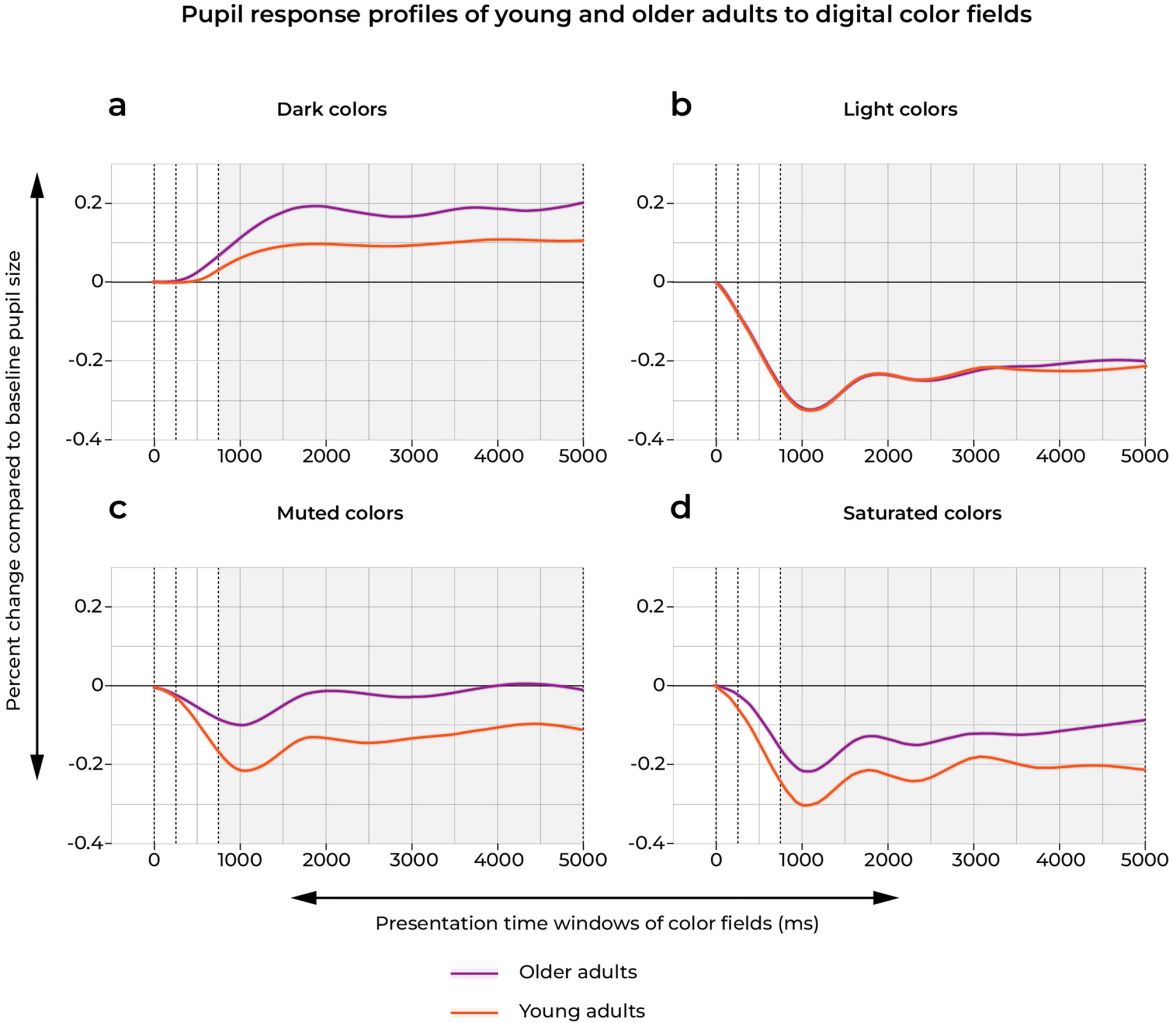
Pupil response profiles relative to baseline of young and older adults plotted against the duration of the stimulus presentation (5000 ms) at a sampling rate of 100 Hz, and parsed in 3 time windows aligning with distinct processing phases of aesthetic stimuli: 0–250 ms; 250–750 ms; 750–5000 ms. Only the recorded pupil data during the 750–5000 ms time window, indicated by the shaded areas in the graphs, were used in the linear mixed models (see further detailed under the subsection Data Analyses under “Materials and methods”). Pupil responses to the color stimuli have been grouped by modifications in color lightness (dark vs light) and colorfulness (saturated vs muted)—See also Fig. 5.

To illustrate the pupil dynamics of young and older adults in response to the color fields, Fig. 2 shows the pupil response profiles relative to baseline pupil size of young and older adults. The pupil responses to color fields have been grouped by modifications in either color lightness (dark vs light) or colorfulness (saturated vs muted) and the slopes show the averaged pupil responses of young and older adults over the duration of the color presentations (5000 ms). The pupil responses were recorded continuously, and afterwards segmented in three time windows: 0–250 ms; 250–750 ms; 750–5000 ms, which are thought to correspond with 3 distinct processing phases of aesthetic stimuli. Only the pupil data that were recorded during the 750–5000 time window were used in the linear mixed models described below (see for further details the subsection “Data analysis” under “Materials and methods”).

To investigate the effects of color lightness and chroma on pupil responses in young and older adults, a series of linear mixed effects models were fitted to the normalized pupil responses with a random intercept for each individual participant and for each color stimulus. The normalized pupil response was calculated as the log of the ratio of average pupil size under experimental condition (recorded between the 750–5000 ms time window after color stimulus presentation), to pre-trial baseline pupil size (averaged over the recorded 5000 ms–1000 ms time window before color stimulus presentation). This was scaled to have mean zero. The ratio was taken in order to account for differences in baseline pupil size between individuals; the log was taken to reduce the skew which would have made modelling with the assumption of normality of residuals difficult. A random intercept at the level of the individual participant was included in all models. Two models were fitted to the pupil responses, which included the following main fixed effects: lightness, cohort (young vs older adults), sex, index order, as well as:

MODEL 1: Chroma as a measure of saturation (colorfulness), defined as the C* value in the CIELCh color space.

MODEL 2: Statistical interactions between the four CIELAB polar hue dimensions (Green, Magenta, Blue, and Yellow) and cohort.

The formula we used for Model 1 to derive the chroma (relative saturation/colorfulness) values from the CIELAB a* and b* coordinates of the all color stimuli was:

$${\text{C*}} = \surd ({\text{a*}}^{2} + {\text{b*}}^{2} ).$$

The term ‘interaction’ in the definition of Model 2 refers to a statistical interaction term; that is, two terms multiplied together and included in the regression model. The CIELAB polar hues are defined by the a* coordinate (Green opposite Magenta) and the b* coordinate (Yellow opposite Blue). For Model 2, we derived 4 separate polar hue variables from the CIELAB a* and b* coordinates, transforming the negative a* and b* coordinates (indicating relative Green and Blue saturation respectively) into positives values. For instance, the CIELAB a*, b* coordinates of the Saturated Yellow color stimulus were: a* = − 5.36, b* = 88.10. We transformed the a* and b* coordinates into four CIELAB polar hue values as follows: Green = 5.36, Magenta = 0, Yellow = 88.10. Blue = 0. The CIELAB polar hue values of each color stimulus are detailed in Table 3.

### Pupil responses to color lightness and chroma in young and older adults

**Figure 3:**
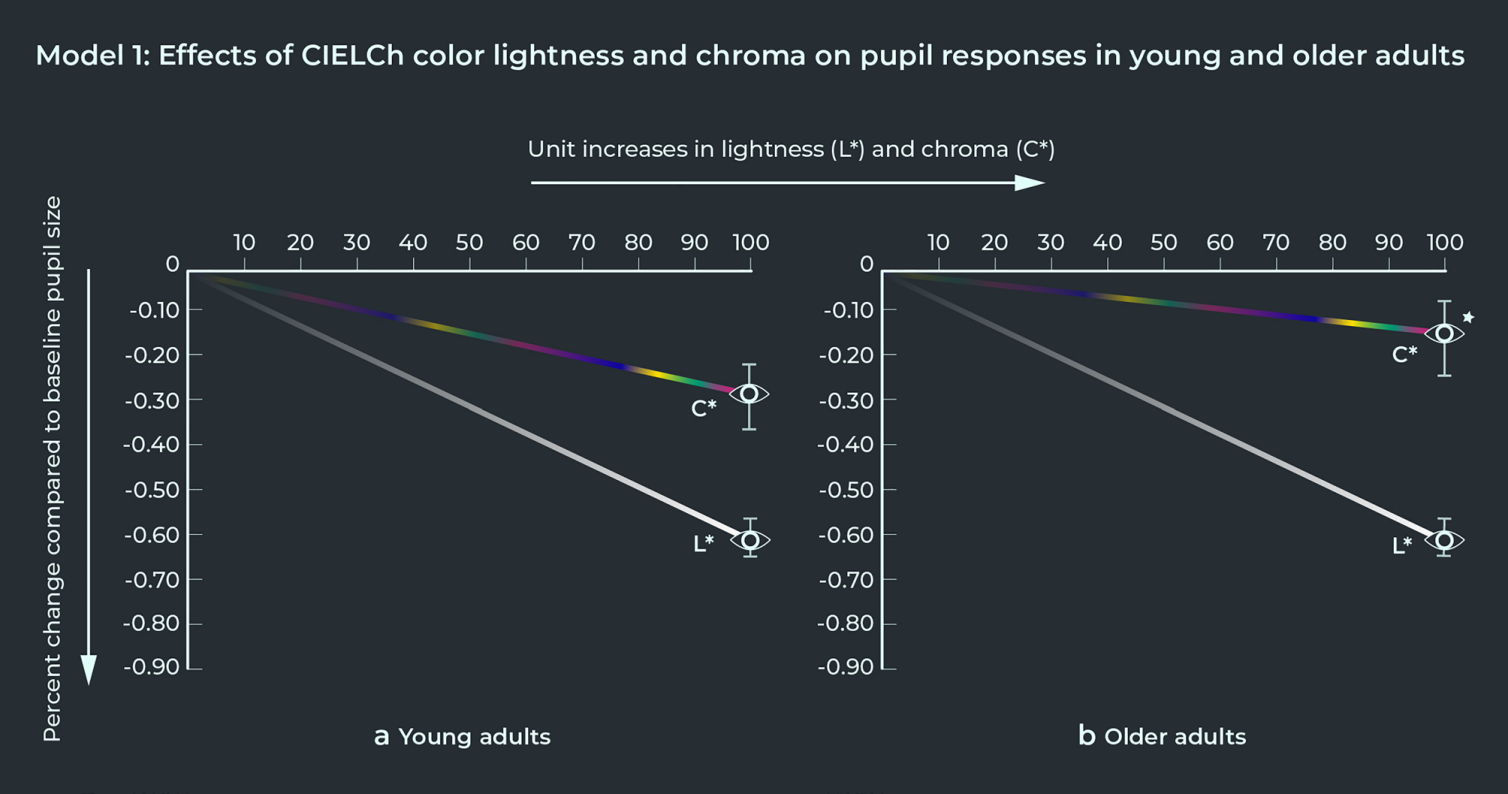
Pupil responses to color lightness (L*) and chroma (C*) in young and older adults, defined in the CIELCh color space. Sustained pupil response to the color stimulus was calculated as the log of the ratio of average pupil size in the right eye under experimental condition, to pre-trial baseline right-eye pupil size. The vertical markers on each eye symbol indicate the confidence intervals of the percent change compared to baseline pupil size. (**a**) Shows the pupil responses to increasing levels of lightness (L*) and chroma (C*) in young adults. (**b**) Shows the pupil responses to increasing levels of lightness (L*) and chroma (C*) in older adults. Older adults’ pupil responses to increases in chroma (C*) were significantly weaker compared to young adults (statistical significance indicated with ).

MODEL 1 showed there is strong evidence for main effects of color lightness (L), chroma (C), and cohort on sustained pupil responses to broad-spectrum colors. Color lightness had the largest effect on the pupil response, with no difference between young and older adults (Table 1). MODEL 1 showed that at a maximum color lightness level in the CIELCh color space (L = 100), pupil sizes of both young and older adults were approximately 61% smaller relative to baseline (Fig. 3). There was no evidence of an interaction between color lightness and chroma, suggesting that color lightness and chroma have independent effects on pupil responses. There was strong evidence of an interaction effect between chroma and cohort however, indicating that pupil responses to the colorfulness of broad-spectrum colors are weaker in older adults. At a maximum chroma (colorfulness) level in the CIELCh color space (C = 100), pupil sizes of young adults were approximatively 29% smaller relative to baseline, compared to approximately 17% smaller pupil sizes relative to baseline in older adults (Fig. 3).

**Table 1:** MODEL 1: Pupil responses to the digital broad-spectrum color fields, including pairwise two-way interactions between lightness, chroma (colorfulness) -as defined by the L and C values in the CIELCh color space-, and cohort (young vs older adults), as well as the following covariates including: sex, and color stimuli index.

MODEL 1 Pupil responses to broad-spectrum colors			
Predictors	Estimates	Confidence intervals	P values
Main effects			
(Intercept)	0.937	0.870–1.009	0.083
Lightness (L*)^i^	0.910	0.900–0.922	** < 0.001**
Chroma (C*)^ii^	0.975	0.965–0.985	** < 0.001**
Cohort: Young adults	*Reference*		
Cohort: Older adults	1.106	1.057–1.158	** < 0.001**
Sex: Male	*Reference*		
Sex: Female	0.994	0.950–1.041	0.808
Index	0.999	0.995–1.003	0.746
Interaction terms			
Lightness by chroma	1.000	0.996–1.004	0.900
Lightness by cohort: young adults	0.910	0.899–0.922	0.843
Lightness by cohort: older adults	0.911	0.899–0.922	
Chroma by cohort: young adults	0.966	0.955–0.976	** < 0.001**
Chroma by cohort: older adults	0.982	0.971–0.992	
Random effects			
σ^2^	0.02		
τ_00 ID.code_	0.00		
τ_00 Color.label_	0.00		
ICC	0.32		
N_ID.code_	37		
N_Color.label_	26		
Observations	782		

The pupil responses were computed as the log of the ratio of pupil size under experimental condition to baseline pupil size. Random effects added to the model included individual participant pupil data (ID code), color label, the total number of color trials (Observations), and the ratio between marginal regression coefficients and conditional regression coefficients. Main effects were found of color lightness (L), chroma (C ) and cohort. An interaction effect between chroma and cohort was found as well, which indicated a significant difference between the pupil responses to color saturation between young and older adults, whereby pupil responses to chroma intensity in older adults were weaker (see also Fig. 3).

^i^Effect averaged over cohort and with chroma (saturation) held constant at C= 50 in the CIELCh color space.

^ii^Effect averaged over cohort and with lightness held constant at L = 50 in the CIELCh color space.

### Two-way interaction effects of cohort and CIELAB polar hue saturation levels on pupil responses

**Figure 4:**
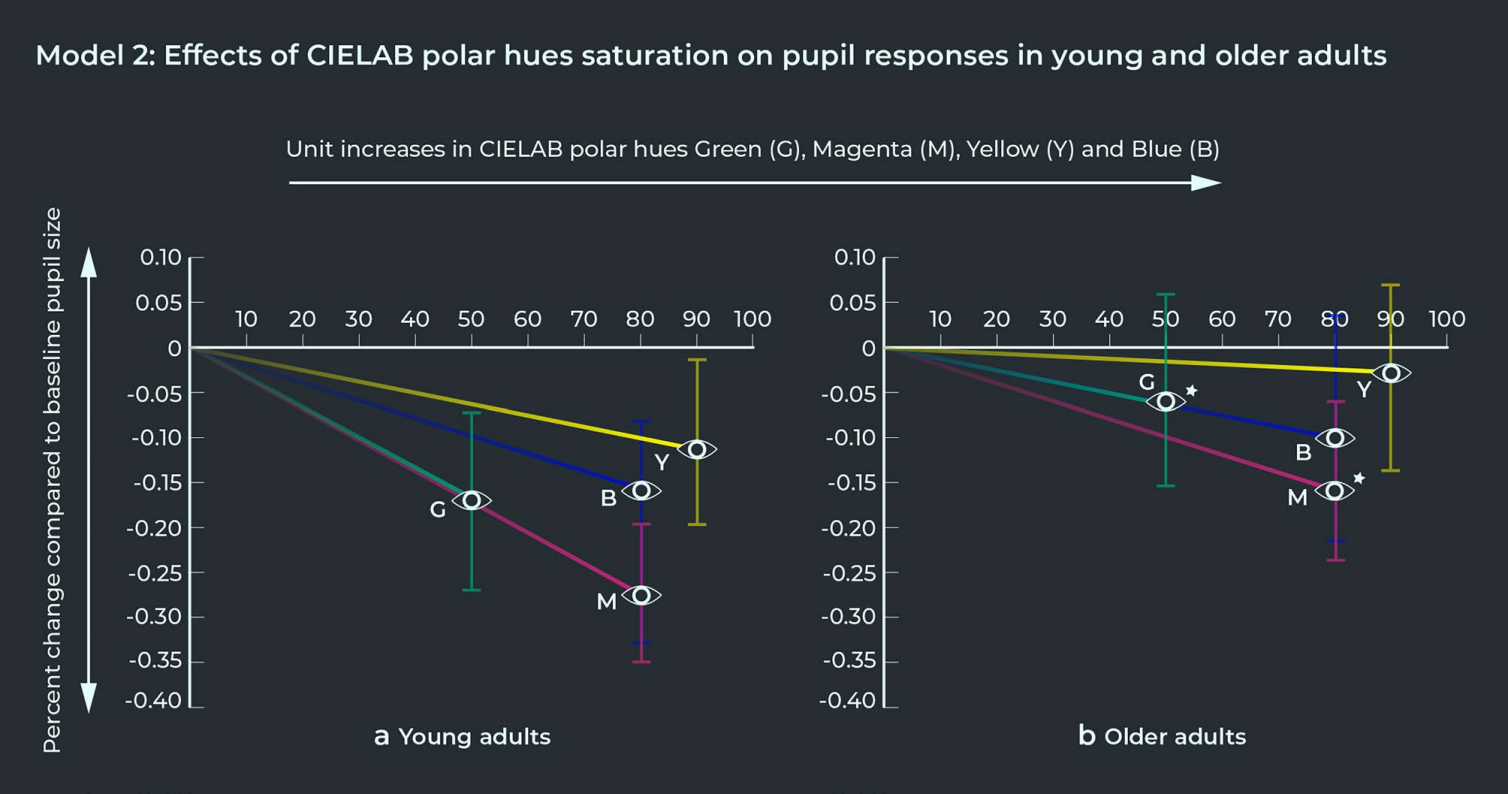
Pupil responses to increases in saturation of the four polar CIELAB hues (Green, Magenta, Yellow, and Blue) in young and older adults, plotted to their maximum saturation level in the color selection. Sustained pupil response to the color stimulus was calculated as the log of the ratio of average pupil size in the right eye under experimental condition, to pre-trial baseline right-eye pupil size. The vertical markers on each eye symbol indicate the confidence intervals of the percent change compared to baseline pupil size. (**a**) Shows the pupil responses to increases in relative saturation levels of Green, Magenta, Yellow, and Blue in young adults, with relative Green or Magenta saturation levels having stronger effects than relative saturation levels of Blue or Yellow. (**b**) Shows the pupil responses to increases in relative saturation levels of Green, Magenta, Yellow, and Blue in older adults, with significantly weaker constriction responses—and a range of responses that also include pupil dilations—to relative Magenta or Green saturation levels compared to young adults (statistical significance indicated with ). In older adults, relative Magenta saturation level had the strongest effect on pupil responses, while relative Green saturation appeared to have a similar effect as relative Blue saturation on pupil responses in older adults.

A second mixed-effect model (MODEL 2) was fitted for the pupil responses to the 26 broad-spectrum color fields to analyze the two-way interaction effects between the four CIELAB polar hues (Green, Magenta, Yellow, and Blue) and cohort (young versus older adults). The results are presented in Table 2 and show that the pupil responses in older adults were significantly weaker than young adults for increases in relative saturation level of Green or Magenta, and this appeared to drive the main interaction effect between chroma and cohort found in MODEL 1. There was a borderline meaningful interaction between cohort and saturation levels of Yellow. Pupil responses to increases in relative Blue saturation were the same in young and older adults on the other hand. Figure 4 shows the pupil responses to saturation increases of the CIELAB polar hues Green, Magenta, Yellow, and Blue in young and older adults. The slopes of the four polar hues have been plotted to their maximum value in the experimental color selection. In young adults, the effect of relative Green or Magenta saturation on pupil constrictions were the strongest and of similar strength. The effects of relative Blue or Yellow saturation on pupil constrictions in young adults were weaker, whereby relative Yellow saturation had the smallest effect on the pupil response. Older adults exhibited significantly weaker pupil constriction responses—and a range of responses that also included pupil dilations—to relative saturation increases in Green or Magenta saturation compared to young adults. At their maximum saturation level within the color selection, pupil constriction responses to Green or Magenta were approximately 11% and 12% smaller in older adults, compared to young adults. While pupil constrictions in response to relative Yellow saturation increases also appeared to become weaker (and include dilation responses as well) in older adults, this difference was only borderline significant in our analysis (approximately 8%). The relative Magenta saturation level had the strongest effect on pupil constriction responses in older adults, while the relative Green saturation level had a similar effect on pupil responses as relative Blue saturation.

**Table 2:** MODEL 2: Two-way interaction effects of cohort and hue saturation on pupil responses with color lightness held constant (L * = 50).

MODEL 2: Two-way interaction effects of cohort and hue saturation on pupil responses			
Predictors	Estimates	Confidence intervals	Joint p values
Magenta: young adults	0.961	0.948–0.974	**0.001**
Magenta: older adults	0.979	0.966–0.992	
Green: young adults	0.963	0.94–0.986	**0.003**
Green: older adults	0.988	0.966–1.011	
Blue: young adults	0.979	0.961–0.997	0.225
Blue: older adults	0.987	0.97–1.005	
Yellow: young adults	0.987	0.975–0.999	0.054
Yellow: older adults	0.996	0.984–1.008	

Results indicate there were significant interactions between cohort and relative saturation intensity of the CIELAB polar hues Green and Magenta, with older adults showing weaker pupil responses compared to young adults to increases in relative saturation of Green or Magenta, and to a lesser degree Yellow (bordering on statistically significant). The effect of relative Blue saturation levels on pupil responses were the same for young and older adults.

## Discussion

In summary, our findings show that older adults exhibit weaker pupil constriction responses to increases in chroma (colorfulness) levels of broad-spectrum colors, compared to young adults. This is particularly the case for colors with relatively high saturation levels of Green or Magenta (described by the a*coordinates in the CIELAB color space). Pupil responses to increases in relative Blue or Yellow saturation (described by the b* coordinates in the CIELAB color space), and the lightness level of colors (described by the CIELAB L coordinate) appear to be unaffected by healthy ageing on the other hand, although we did find a borderline significant effect for Yellow.

Our pupillometry data suggest that we become physiologically less sensitive to the colorfulness of our environment as we age. These findings complement earlier behavioral research which showed that older adults perceive surface colors as less chromatic (colorful) than young adults^12,13^. We therefore propose that colors fade with age, and that we become specifically less sensitive to the relative Green or Magenta saturation level of colors. Our findings show no reduced pupil responses to relative Blue saturation level of colors. The b* coordinate of the CIELAB color space—which codes for relative Blue saturation—, does not map linearly onto the retinal S-cone wavelength sensitivity, but this finding does cast further doubt on the assertion that older adults become less sensitive to blue light^13,17–22^.

The selective effect of healthy aging on pupil responses to color chroma levels might indicate that color lightness and chroma processing are underpinned by distinct neural mechanisms. It seems plausible that the pupil response to color lightness is predominantly regulated by the Pupillary Light Reflex circuitry in the mid-brain^29^. If the observed weaker pupil response in older adults to relative saturation levels of Green or Magenta (defined in the CIELAB color space) in broad-spectrum colors were driven by a decline in the sensitivity of the L and M cone photoreceptors, and/or the signal transmission in the parvocellular pathway, we would also expect to see a selectively weaker pupil response to the lightness levels of colors with high levels of Green or Magenta. However, our data did not show this. We therefore infer that the selective decline in sensitivity to chroma (colorfulness) in older age is more likely rooted in higher cortical areas within the visual pathway. We will next elaborate on the possible mechanism and neural correlates that might underly this.

The development of new theoretical and computational models that ground human color experiences in higher cortical processes is a topic of active interest^2,51–53^. Color-sensitive neurons in the primary visual cortex (V1) have been shown to respond to a wide range of colors, and the majority of these cells are also responsive to variations in luminance and dynamic spatial signals. V2 has also been found to be highly responsive to color information and is thought to receive mixed input from magno-, parvo- and koniocellular pathways in two broad streams, originating from cytochrome oxidase patches and interpatches in V1^54–56^. Research has found a close alignment between the CIELAB perceptual color space and color-tuned glob cells in the extrastriate cortical area V4 in macaque^51^, as well as color preferences in humans^57,58^. Cerebral achromatopsia—an inability to perceive colors due to brain damage in the occipital cortex—has been associated with damage to the V4 area^59,60^, and it has been dubbed the color area of the macaque (and by extension, human) brain^61,62^. Zeki and Marini^63^ proposed that the V4 area is specifically responsible for color constancy; the ability to perceive objects in the same color under varying lighting conditions. However, the claim that V4 is the most important brain area for macaque and human color perception is now largely debunked. Selective damage to the extrastriate V4 has been shown to only partly impact color perception in macaque, and the key importance of V1 and V2 to human color perception—including color saturation—has been extensively documented^4,42,54^.

Low-level pupil (bottom-up) responses are mainly driven by retinal illuminance and tend to be sustained, whereas intermediate and higher cortical (top-down) attentional/orientating pupil responses are more transient in nature^64^. The dynamics of the measured pupil responses in our study (Fig. 2) show that the pupil responses to modulations in color lightness and chroma both resulted in sustained pupil responses during the entire presentation window of 5 s. With the exception of pupil responses to muted (desaturated) colors in older adults however, which returned close to baseline during the 750–5000 ms time window. This could be indicative of a decline in physiological sensitivity to chroma (colorfulness), but it could also signal a higher-order attentional dulling to the effects of chroma.

Aside from possible top-down attentional influences, we believe that a decline in physiological sensitivity to the saturation levels of colors (specifically their relative Green or Magenta intensity) within the primary visual cortex (V1) in older adults could at least partly explain our findings. Below we will briefly elaborate on relevant research that together with the findings of this study informed our position on this.

The Helmholtz-Kohlrausch effect denotes the phenomenon that strongly saturated colors are perceived as brighter by the human visual system, compared to colors with the same luminance but lower saturation^65^. The Helmholtz-Kohlrausch effect has been demonstrated in observer judgements of perceived surface reflectance and color brightness^66,67^. Suzuki et al.^68^ found a correlation between perceived color brightness and the magnitude of pupil constriction, and Xing et al.^11^ reported brightness-color interactions in the primary visual cortex (V1) by means of recording the chromatic visual-evoked potential. In line with these findings, Corney et al.^69^ have proposed that the anatomical base for the Helmholtz-Kohlrausch effect is likely to be situated in V1 and not earlier in the visual processing trajectory, based on a Bayesian ideal observer model of the human visual ecology, combined with fMRI brain scans. Of further relevance to our study is the fact that the Helmholtz-Kohlrausch effect is not compensated for by the CIELAB perceptual color space^70^.

Further evidence for a key role of the primary visual cortex in the appearance of colorfulness comes from a recent study by Shir et al.^50^, which reported a primary occipital variant of posterior cortical atrophy, a rare form of dementia which primarily affects visuospatial functions in early disease stages. The authors found no significant differences in higher-order object and space perception between the different PCA phenotypes included in the study, but the occipital variant of PCA was found to be associated with poor performance on the Ishihara test for color perception, which is indicative of an acquired Red—Green color-blindness due to neurodegeneration of the visual cortex. While the Ishihara test is more difficult to interpret in PCA due to the confounding influence of visuospatial integration deficits, other studies that used different methods have also reported diminished color perception abilities in PCA. A study by Lehmann et al.^71^ found that color discrimination deficits in PCA were associated with higher-order object and space perception, contrary to the findings of Shir et al.^50^. A case report by Chan et al.^72^ described a patient with PCA, who in addition to a reduced chromatic sensitivity, experienced pronounced color afterimages of abnormal latency, duration, and amplitude after exposure to strong color stimulation. These deviations in color processing caused objects to look unnaturally colored. For example, the patient perceived her hands as looking green after putting on red bedsheets. Given that this case had relatively focal atrophy involving posterior occipital cortex, the authors argued that the color perception problems of this patient likely originated in V1. In summary, the pronounced reductions and aberrations in color perception that are observed in PCA could perhaps be the result of an aggravated decline in (Green–Magenta) chroma sensitivity in V1 and connected occipital networks that regulate the appearance of colorfulness, which would be in line with our findings.

While our pupillometry data show there is evidence for a significant decline in the physiological sensitivity to relative Magenta saturation in older age, Magenta still elicited the strongest pupil constriction response in older adults of all the four polar CIELAB hues (Green, Magenta, Blue, and Yellow). This might suggest that colors with relatively high Magenta saturation levels appear as the most colorful to older adults, especially when combined with a high color lightness level (e.g., the color Fuchsia). Relative Green and Magenta levels elicit a pupil response of similar strength in young adults, but in older adults the pupil response to relative Green saturation levels was of similar strength as the pupil response to relative Blue saturation levels (see Fig. 4). This shift offers a candidate explanation for psychophysical studies which found that older adults perceive ‘unique Green’ to be closer to the blue spectrum than young adults^13,18,19^.

Further research is warranted to test our hypothesis that V1 becomes less sensitive to chroma (colorfulness) in healthy aging. Delineating the neural correlates of the multiple components (hue, lightness and chroma) of real-world color appearance in healthy and pathological aging will hopefully also shine a light on how a reduced sensitivity to relative Green and Magenta saturation in older adults might relate anatomically and physiologically to cortical Red—Green color-blindness accompanying later-life neurodegenerative disorders of the visual cortex. This is also of clinical relevance. Emerging evidence suggests that environmental visual cues may facilitate navigation, object detection, and task performance in people with dementia who experience visuoperceptual difficulties^73,74^. Usually, colors with high salience or strong contrast enhance patient performance. However, the findings from this study, combined with insights gained from research into PCA-related color perception decline^50,71^, suggest that in older populations with neurodegenerative conditions affecting the occipital cortex, a thorough color perception assessment should be made to ensure that the use of visual cues has the desired therapeutic effect. Such an assessment should determine both color discrimination abilities as well as the perceived colorfulness of different colors with varying degrees of lightness and saturation. Similar considerations might apply when designing environments and other applications relying on color perception for the healthy elderly. Failing to appropriately tailor visual cues to both color discrimination ability and saturation sensitiveness of the individual might give rise to misleading conclusions about the effectiveness of interventions.

A limitation of this study is that we did not include a behavioral measure on the perceived saturation (colorfulness) of the color selection to collate with the pupillometry data. However, as detailed under “Materials and methods”, all participants were able to distinguish between the ‘Saturated’, ‘Muted’, ‘Dark’, and ‘Light’ color manipulations that were made across the color categories ‘Greyscale’, ‘Purple’, ‘Blue’, ‘Green’, ‘Yellow’, and ‘Red’. It is also interesting to consider our findings in relation to the popular folk belief that older people develop a preference for beige clothes. But as Jenny Joseph intuited in her poem ‘Warning’^75^, it might be equally likely that in later life, people develop a strong preference for intense colors such as purple and red, perhaps as compensation for the reduced physiological sensitivity to chroma. Future research into the dynamics between the physiological, psychological, and behavioral changes related to color appearance in healthy aging and neurodegenerative disease will hopefully further illuminate this.

## Materials and methods

### Participants

Thirty-seven research participants with no history of neurological illness were recruited via public social media, as well as via internal communication platforms at the Wellcome Collection and the UCL Dementia Research Centre in London, where the study jointly took place. The study received ethical approval by the University College London Research Ethics Committee (8545/002: Created Out of Mind) and the UCL Queen Square Research Ethics Committee (17/LO/0099). All research methods were performed in accordance with relevant guidelines and regulations.

Seventeen young adults were recruited (Female = 7, μ = 26.7 years) and twenty older adults (Female = 10, μ = 64.4 years). Prior to inclusion in the study, informed consent was obtained from all participants. Participants filled out the first two sections from the UCL Dementia Research Centre demographics questionnaire, which concerned questions on personal background and general health. An added third section gathered information on how much experience participants had regarding the practical and theoretical aspects of visual art. Most participants were right-handed (94% young adults; 80% older adults, an insignificant between group difference) and there was a comparable distribution of education levels and art experience between the young and older adult cohorts. None of the participants had a history of neurological conditions or visual impairments (including cataracts), which were exclusion criteria. Corrective lenses were allowed, as long as they were not tinted and it was possible to calibrate the eye tracking camera, which was the case for all participants. General visuospatial cognitive abilities were assessed with an abbreviated version of the Wechsler Abbreviated Scale of Intelligence (WASI) Matrix Reasoning (Wechsler, 1999), a standardized test which aims to measure perceptual reasoning ability. The test consisted of 18 multiple choice items increasing in difficulty which were presented on an iPad, every correct answer equaled 1 point. The mean score of the young adults cohort was 16.3 (SD = 1.0) and the mean score of the older adults cohort was 15.5 (SD = 1.8), both cohorts performed within the normal range and the group mean difference was insignificant.

All participants had a 100% percent score on the Matching Colors Scale, a novel 12-item color perception task designed as a tight low-level visual perceptual control task to test basic color perception. The scale assessed participants’ ability to perceive variations of lightness and saturation within each color category of the experimental color stimuli (Purple, Blue, Green, Yellow, Red and Greyscale), as well as between colors of similar saturation or lightness belonging to different color categories. The rationale behind creating a novel instrument, rather than choosing an existing color perception test, was that this allowed us to align the color items exactly with the color selection of the experimental tasks. The test design of the Matching Colors Scale is further detailed in the legend of Supplementary Fig. 1, which shows the 12 items of the Matching Colors Scale with alphabetical letters indicating the order in which the items were shown to the research participants.

### Color stimuli

**Figure 5:**
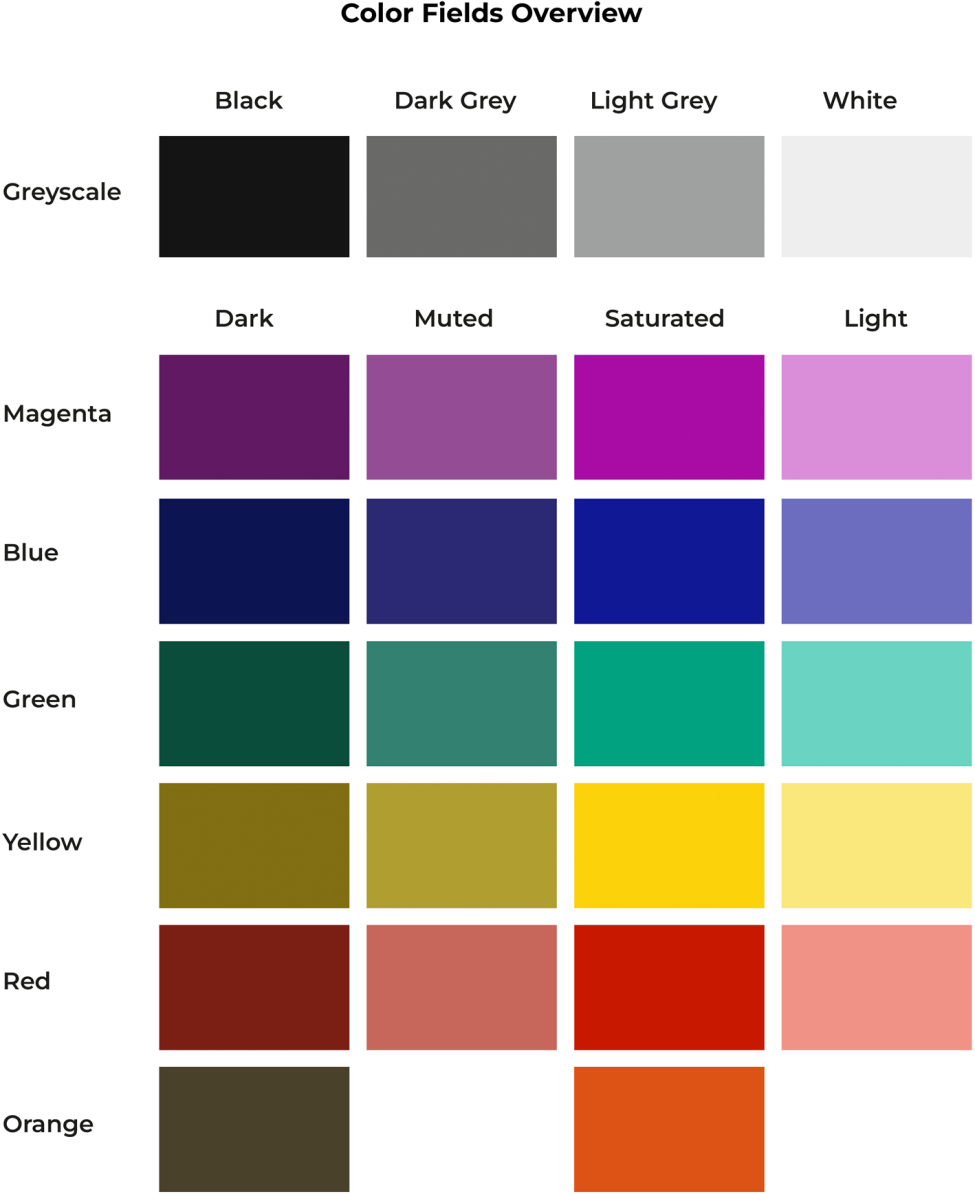
Overview of the 26 broad-spectrum color fields used as experimental stimuli in the pupillometry experiment.

A set of 26 digital color fields (Fig. 5) was created using the Pantone Matching System (PMS) color system. The color selection and experimental design were part of a larger study into the physiological and psychological responses to colors in different spatial and material presentations. The advantage of the Pantone system is that it defines the color properties across both digital and print media, which facilitates accurate color-matching between digital and print stimuli.

Four Greyscale colors were chosen: Black (5% lightness); Dark Grey (45% lightness); Light Grey (65% lightness); and White (95% lightness). These were complemented with the following five Saturated Hues: Purple; Blue; Green, Yellow; and Red from the Pantone Color Bridge Coated Guide. To create variations in lightness and chroma of the color stimuli, for each of the five selected Saturated hues, a Muted, Dark, and Light modification was made in Adobe Photoshop CC (2017). A Hue/Saturation layer was added to the Saturated Purple, Blue, Green, Yellow and Red color swatches, which was set to normal blending mode. To create the Muted variant of each Saturated hue, the Hue/Saturation layer was set to − 50% in the Saturation channel. To create the Light variant of each Saturated hue, the Hue/Saturation layer was set to + 50% in the Lightness channel and to create the Dark variant of each Saturated hue, the Lightness channel was set to − 50%. Adobe’s color management is based on conventions developed by the International Color Consortium (ICC) standards^76^. This means that the color modification algorithms in Photoshop are tailored to perceptual color consistency within the defined color space, rather than creating alterations of equal size for each color manipulation. Therefore, while the color stimuli of this study were perceptually balanced across the four color manipulations ‘Saturated, Muted, Dark and Light’ in Photoshop, their corresponding CIELAB values were not equally distributed across the color space. Two additional colors were added to the selection: The supposedly ‘ugliest color’ in the world, Pantone 448C^77^ as well as a Saturated Orange on the opposite end of the brightness spectrum compared to Pantone 448C.

The color lightness and chroma values of the color stimuli (N = 26) were defined in CIELCh, a vector representation of the CIELAB color space^78^. The CIELAB perceptual color space (Supplementary Fig. 2) is a device-independent, "standard observer" model of human color perception^79^. In this three-dimensional color space, color lightness (L*) is defined in the vertical plane and the horizontal plane describes the combined hue and relative color saturation values, whereby the a* coordinate codes for the relative Green or Magenta saturation, and the b* coordinate codes for the relative Blue or Yellow saturation. While Green and Magenta are each other’s complementary color—which is also evident in color afterimages—the positioning of Green opposite of Magenta in the CIELAB color space (defined by the a* coordinate) has been criticized by some. Seymour^80^ has argued that the CIELAB polar hues should align more closely with the retinal cone functions, and that Red should be coded opposite Green by the a* coordinate instead of Magenta. Regardless, it has been shown that indices of color saturation (defined by the a* and b* coordinates) in the CIELAB color space align accurately with the human perception of how colorful colors appear^81^. In the CIELCh color space, L* indicates the lightness, C* indicates the chroma (colorfulness), and h indicates the hue angle within a 360 degrees distribution of the visible light spectrum (Supplementary Fig. 2).

The concepts chroma and saturation both indicate the degree of colorfulness, but these two concepts are not always equal in every circumstance. The definition of chroma in the CIELCh color space (derived from the CIELAB a* and b* coordinates) refers to the radial position in the horizontal plane, whereby the more distal coordinates indicate a higher level of colorfulness. Color saturation is calculated in proportion to the reference white point, and perceived saturation is influenced by contextual luminance and color contrast. However, we argue that in our study chroma is a reasonable indicator of relative saturation for the following reasons:

- The reference white point (D65), and the monitor luminance (100 cd/m^2^), background color (black) and contrast were kept constant throughout the experiment (see detailed under the subsection Apparatus under “Materials and methods”, page 21), which controlled for any confounding influence of contextual luminance or color contrast on perceived color saturation.
- Model 1 showed no statistical interaction effect between color lightness and chroma, meaning that the combinations of lightness and chroma values of the color stimuli did not have an additional effect on pupil responses, which is in line with the perceptually linear nature of the CIELAB color space. Perceptually linear in this context means that a change of the same amount in a color value should produce a change of about the same visual importance.

The CIELAB lightness (L) and the a* and b* coordinates were read out from the color swatches in Photoshop.

The chroma (C) values were computed by converting the CIELAB values to the CIELCh color space using an online calculator^82^, using D65 as reference white point. This resulted in a data set of 26 color stimuli varying in hue, lightness, and saturation, whereby each color stimulus was defined by a chroma and lightness value, as well as a saturation value of the polar hues Green, Magenta, Yellow, and Blue. The chroma, lightness and polar hue values fell within a broad range of integer values between 0 and 100.

Table 3 shows an overview of the color data of each color stimulus.

**Table 3:** Color Data.

Color label	R	G	B	HEX #	Lightness L*	Chroma C*	a* M+/G−	b* Y+/B−	Magenta	Green	Yellow	Blue
Dark Black	18	18	18	121212	5.46	0.00	0.00	0.00	0.00	0.00	0.00	0.00
Dark Blue	8	3	80	80350	6.29	54.21	31.57	− 44.07	31.57	0.00	0.00	44.07
Dark Brown	74	65	42	4A412A	27.86	15.46	− 0.04	15.46	0.00	0.04	15.46	0.00
Dark Green	0	85	67	005543	31.58	27.79	− 27.47	4.21	0.00	27.47	4.21	0.00
Dark Grey	82	82	82	525252	34.88	0.01	0.01	0.00	0.01	0.00	0.00	0.00
Dark Purple	94	21	94	5E155E	22.74	49.21	41.66	− 26.20	41.66	0.00	0.00	26.20
Dark Red	125	28	17	7D1C11	27.38	51.76	40.69	32.00	40.69	0.00	32.00	0.00
Dark Yellow	127	111	0	7F6F00	46.82	53.14	− 3.98	52.99	0.00	3.98	52.99	0.00
Light Blue	135	130	207	8782CF	57.68	43.93	19.96	− 39.13	19.96	0.00	0.00	39.13
Light Green	127	212	194	7FD4C2	79.32	29.95	− 29.94	0.81	0.00	29.94	0.81	0.00
Light Grey	159	159	159	9F9F9F	65.49	0.01	0.01	0.00	0.01	0.00	0.00	0.00
Light Purple	221	148	221	DD94DD	70.71	46.88	38.97	− 26.05	38.97	0.00	0.00	26.05
Light Red	252	155	144	FC9B90	73.64	40.92	35.01	21.18	35.01	0.00	21.18	0.00
Light White	243	243	243	F3F3F3	95.84	0.02	0.02	− 0.01	0.02	0.00	0.00	0.01
Light Yellow	254	238	127	FEEE7F	93.34	55.85	− 8.68	55.17	0.00	8.68	55.17	0.00
Muted Blue	49	44	120	312C78	23.03	50.06	25.82	− 42.89	25.82	0.00	0.00	42.89
Muted Green	42	127	109	2A7F6D	47.98	29.95	− 29.86	2.38	0.00	29.86	2.38	0.00
Muted Purple	150	77	150	964D96	44.22	49.43	41.42	− 26.97	41.42	0.00	0.00	26.97
Muted Red	195	99	88	C36358	53.20	44.30	37.28	23.92	37.28	0.00	23.92	0.00
Muted Yellow	190	173	63	BEAD3F	70.31	56.92	− 6.69	56.52	0.00	6.69	56.52	0.00
Saturated Blue	16	6	159	10069F	18.99	91.64	54.06	− 74.00	54.06	0.00	0.00	74.00
Saturated Green	0	179	138	00AA86	65.02	49.44	− 48.36	10.24	0.00	48.36	10.24	0.00
Saturated Orange	223	83	21	DF5315	58.80	94.20	63.64	69.45	63.64	0.00	69.45	0.00
Saturated Purple	187	41	187	BB29BB	46.63	84.29	71.45	− 44.71	71.45	0.00	0.00	44.71
Saturated Red	249	56	34	F93822	55.16	90.70	70.38	57.21	70.38	0.00	57.21	0.00
Saturated Yellow	254	221	0	FEDD00	88.34	88.26	− 5.36	88.10	0.00	5.36	88.10	0.00

The first column describes the color label of each color in the color selection of the study (N = 26). The RGB values were defined in the sRGB IEC61966-2.1 color space. The hex codes are the web codes for each color in the selection. The Lightness L* and Chroma C* values were defined in the CIELCh color space, based on a white point of 6500 K (D65)—the reference white point of the calibrated monitor. These values were used in the statistical analysis, alongside the a*b* coordinate values of the color stimuli as defined within the CIELAB color space. The ‘a*_M+/G− ‘ column shows the a* coordinate values of the color stimuli, in which positive values indicate the relative Magenta saturation, and negative values the relative Green saturation. The ‘b*_Y+/B− ‘ column shows the b* coordinate values of the color stimuli, in which positive values indicate the relative Yellow saturation and negative values indicate the relative blue saturation. The CIELAB a* and b* coordinates were separated out in unipolar positive values across the last four columns in the table, in order to analyze the potential effects on pupil responses of the CIELAB polar hues Green, Magenta, Yellow, and Blue.

### Apparatus

The color pupillometry experiment was programmed and run using the SR Research Experiment Builder software package. The color stimuli were presented on an Eizo ColorEdge CG2420 24-inch LCD monitor, which was placed at 75 cm distance from a table-mounted headrest which stabilized the chin and forehead of the participants. For each participant the height of the chinrest was adjusted so that their eyes aligned with the top 25% of the monitor. The display area of the monitor measured 518.4 × 324.0 mm with a native resolution of 1920 × 1200 (16:10 aspect ratio) and was calibrated with an X-rite Eye One Display 2 device, using Eizo ColorNavigator6 software which was installed on a connected 13-inch late 2016 MacbookPro laptop from which the experiment was run. The target color profile of the Eizo ColorEdge CG2420 24-inch LCD monitor was defined within the sRGB color space at a brightness of 100 cd/m^2^, a white point of 6500 K, and the brightness level of black set to 0.5 cd/m^2^. The tone curve of the monitor was defined at a RGB gamma of 2.2 with a standard priority. The monitor background color behind each color stimulus presentation was black (0.5 cd/m^2^). A SR Research EyeLink 1000 Plus eye tracking camera was placed in front of the monitor at a 55 cm distance from the headrest, with the lens directed at the eyes of the participant. The eye tracking camera was calibrated to each individual participant with the Experiment Builder software, using a 9-point grid. Bilateral pupil diameter was recorded at a frequency of 1000 Hz, which created a very large data set. It has been shown that under normal circumstances, pupil responses in the left and right eye are symmetrical^83^, and to make the data set more manageable we therefore only used the pupil dilation data from the right eye recordings (the dominant eye for most participants) in the data analysis. The SR Research eye tracking software identified the pupil outlines based on the darkest area within the recording grid of the eye tracking camera, which was determined by the refraction pattern of an unobtrusive infrared beam that was emitted by the eye tracking camera. The pupillometry data were recorded onto a Dell laptop, which was connected to the MacbookPro laptop which ran the experiment. Both laptops were placed on a black table which was positioned in a 90 degrees angle to the left of the experiment presentation table. Supplementary Fig. 3 shows the color experiment set-up (during the experiment the ambient light was turned off).

### Procedure

The color pupillometry experiment took place in a blackout room. Research participants were dark-adapted for a minimum of ten minutes. Before the experiment started, a pre-recorded audio instruction explained the procedure of the experiment and participants were given the opportunity to ask questions if anything was unclear. The experiment consisted of twenty-six broad-spectrum digital color fields (1574 × 1050 pixels at 96 dpi), with a visual angle of 31.64 in horizontal direction and a visual angle of 21.41 in vertical direction. Each trial began with a 5-s presentation of a middle grey (18%) screen (1574 × 1050 pixels at 96 dpi) with a fixation cross in the middle to neutralize the pupil dilation and orientate the gaze towards the center of the screen. Then the color stimulus would be presented for 5 s, after which a middle grey screen was presented to participants with a 5-point visual rating scale which asked how the color stimulus had made them feel (1 being strongly positive, 5 being strongly negative and 3 being neutral). After participants had told the experimenter their affect rating, the next trial was started (no time limit). The order of the color stimuli presentation was pseudo-randomized (Supplementary Fig. 4), to ensure no two colors from the same color category (e.g., Light Blue and Dark Blue) were shown in direct succession.

### Data analysis

All analyses were carried out using the R statistical software^84^, using the package lme4^85^. The recorded pupillometry data were pre-processed in Data Viewer, a custom-made software package designed by SR Research, the company that produces the EyeLink eye tracking cameras. Blinks were filtered out in Data Viewer, based on the occurrence of small data gaps in the continuous recordings of pupil dilations. The pupil dilation data points were exported from Data Viewer at a sampling rate of 1000 Hz and parsed in three time windows corresponding with temporal cortical processing phases of aesthetic stimuli, from initial perceptual processing (0–250 ms), to making a gist evaluation of ‘beautiful/ugly’ (250–750), and a deeper processing phase involving higher cortical networks (> 750)^52,86–88^. We were particularly interested in analyzing the pupil responses that corresponded with the cortical processing phase during which conscious processing of colors takes place. For this reason, only the pre-trial baseline pupil recordings and the pupil recordings between the 750–5000 ms interval after presentation were used in the linear mixed model analyses. To this purpose, the pupil responses of each participant to the 26 color stimuli were exported by Data Viewer as a mean pupil dilation (mm^2^), calculated as the sum of all the recorded pupil dilations during the 750–5000 ms time window (at the recording rate of 1000 Hz) per participant, divided by the number of pupil recordings during that time window. Individual average baseline pupil dilations were defined trial by trial, by recording pupil dilations at 1000 Hz during the first 4000 ms of the 5000 ms presentation window of the middle grey fixation screen that was shown before each color stimulus was presented. The pupil recordings in the time window 1000 ms before stimulus presentation were excluded from the baseline pupil analyses, to clearly delineate baseline and experimental pupil measurements.

For the pupil response profiles in Fig. 2, the pupil dilation data were exported from the Data Viewer at a sampling rate of 100 Hz, whereby each pupil data point was accompanied with a recording timestamp. This resulted in a temporally ordered dataset of 100 pupil dilation data points per second, over a total duration of 5 s (the stimulus presentation window), per participant per trial, from which we have plotted the pupil response profiles in Fig. 2. The following statistical method was used to create the pupil response profiles: The first step was to normalise the measurements of the right pupil size. This was done on an individual trial by trial basis by dividing all pupil responses by the baseline pupil size; which was measured at time zero, the moment the baseline period ended and the stimulus exposure began. In order to produce smoothed average pupil response curves, the pupil responses were modelled with a single covariate for time using a generalised additive model (using the function geom_smooth from the R package ggplot). These models were fit separately for each cohort and colour modification group. The fitted curves resulting from these models were plotted with (narrow) 95% confidence intervals.

While the choice of the sampling window of the pupil recordings that we used in the linear mixed effect models was based primarily on the temporal processing phases of aesthetic stimuli, it is also justified with respect to the physiology of the Pupillary Light Reflex and the effects of aging on this. The pupil constriction response has a latency period which has been shown to vary considerably in healthy subjects^89^, with a minimum delay of 180–230 ms^48^. The maximum constriction amplitude of the pupil is furthermore correlated with the size of baseline pupil dilation, and therefore needs to be normalized to baseline pupil dilation^48^. In line with previous research^90–92^, baseline pupil dilations in older adults in this study were significantly smaller compared to young adults. Our pupillometry data controlled for the natural variation in the latency of Pupillary Light Reflect by selecting a sampling window of the pupil responses between 750 and 5000 ms after stimulus presentation. We controlled for the relationship between baseline pupil dilation and the maximum constriction amplitude of the pupil response by computing the log of the ratio of mean pupil size under experimental condition compared to baseline pupil size for each individual study participant.

To be included in the analysis both the baseline and response measurement needed to be present. Given the fact there were 26 color trials and 37 participants, the maximum number of pupil responses would have been 962, defined as the mean pupil dilation (mm^2^) during the 750–5000 ms presentation window of each color trial. Overall, there were 782 pupil responses, meaning that 18.7% of possible pupil responses were missing. A total of 24 individuals had at least one missing response (64.9%); this included 70.6% of young adults and 60% of senior adults. A missing pupil response meant that the eye tracking camera had been unable to record any pupil data (at 1000 Hz) during the entire duration of the color trial. This could for instance be the case if a participant averted or closed their eyes, or if the reflection of their glasses prevented the eye tracking camera from locating the pupil. The median number of missing pupil measurements (baseline and response combined) across all 26 color trials was 20 in the Young Adult cohort (IQR: 10, 22); and 20.5 in the Older Adult cohort (IQR: 15, 22). The Young Adult cohort had slightly more missing pupil data than the Older Adult cohort. In summary, approximately 20% of all pupil responses were missing, but this was within acceptable limits for the statistical analysis method that was used in this study (linear mixed effects models), which can handle missing at random data well by using a maximum likelihood estimation^93^. The fixation patterns of both young and older adults were similar and concentrated around the center of the color field stimuli (Supplementary Fig. 5).

The rationale behind the choice of using linear mixed effect models in this study, in addition to their ability to account for missing data, was that the measurements from the same individual, or the same color stimuli, may be correlated with each other. This violated the assumption of basic statistical models such as regression and general linear models, which assume that the residuals are independently and identically distributed as N (0,σ2) and that residual or unexplainable error is the only source of random variability^93^. Linear mixed effect models can make meaningful inferences from the experimental data when the assumptions of basic statistical models don’t apply. By adding the individual participant responses and the chosen colors in this study as random effects to the models, we were also able to account for any variance in the pupil responses that might be due to interpersonal variability and the particular color selection.

### Supplementary Information

Supplementary Figures.

## Data Availability

The datasets generated and/or analyzed during the current study are not publicly available due to the stipulation of the institutional ethics approvals covering consent and data collection, but are available from the corresponding author on reasonable request.
