# Supplementary material for: Pupil responses to colorfulness are selectively reduced in healthy older adults
Source: Sci Rep. 2023 Dec 13;13:22139. doi: 10.1038/s41598-023-48513-7 (PMC10719259; doi:10.1038/s41598-023-48513-7)
Supplement: Supplementary file 1 — Supplementary Figures. [file 41598_2023_48513_MOESM1_ESM.docx]

**SUPPLEMENTARY MATERIALS**

**Pupil Responses to Colorfulness are Selectively Reduced in Healthy Older Adults (Van Leeuwen et al.)**

**
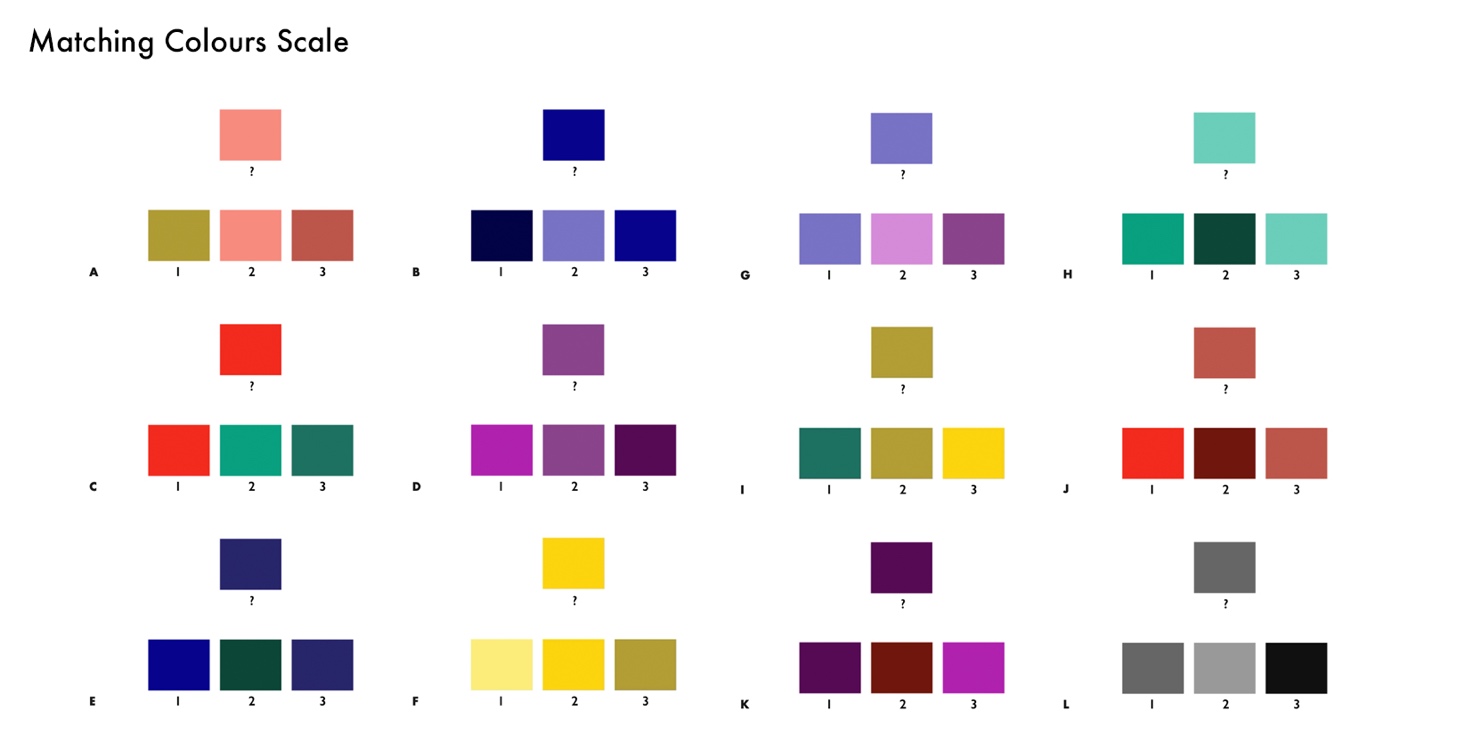
**

**Supplementary Figure 1** Matching Colors Scale, a novel 12-item color perception task designed as a tight low-level visual perceptual control task to test basic color perception. The scale assessed participants’ ability to perceive variations of lightness and saturation within each color category of the experimental color stimuli (Purple, Blue, Green, Yellow, Red and Greyscale), as well as between colors of similar saturation or lightness belonging to different color categories. The rationale behind creating a novel instrument, rather than choosing an existing color perception test, was that this allowed us to align the color items exactly with the color selection of the experimental tasks. Each item consisted of a color block with a question mark placed underneath. In a row below, three numbered color blocks of identical size were shown, with one color block being the exact same hue as the color block with the question mark. Participants were requested to read out the matching color block out loud. Each color category was allocated two items that aimed to assess lightness discrimination in one item and saturation discrimination in the other. The items that aimed to assess lightness discrimination contained at least 1 color block of a neighboring color category on the visible light spectrum among the 3 color block options that participants could choose from. Greyscale perception was assessed in a single item with greyscale color blocks that varied in the same shades of grey as used in the experimental tasks. The 12 items were shown one at the time on an Eizo ColorEdge CG2420 24-inch LCD monitor, which had been calibrated in the sRGB gamut to a white point of 6500K at a brightness level of 100 cd/m² in a dark room.

**
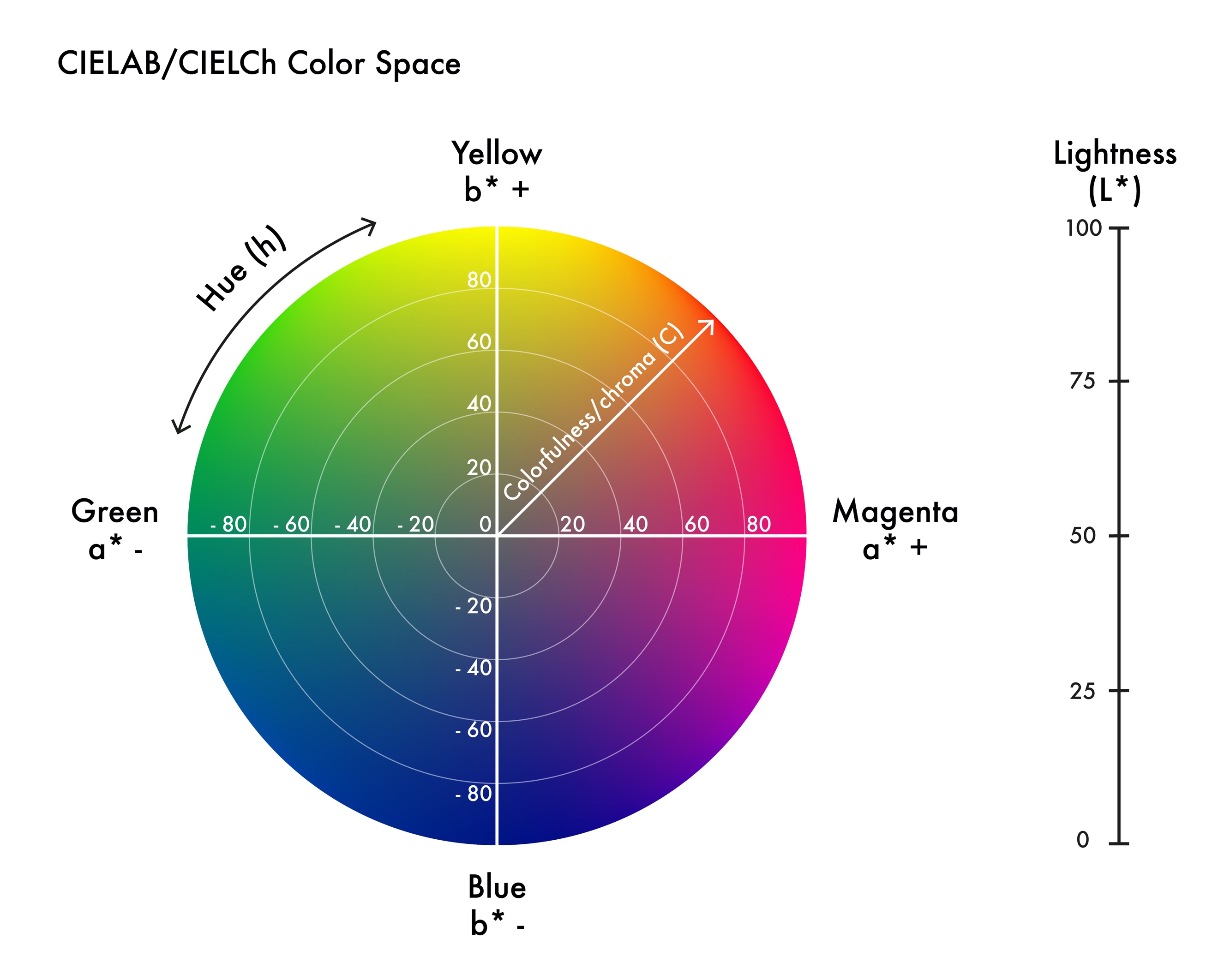
**

**Supplementary Figure 2** Diagram of the CIELAB/Ch perceptual color space. The z-axis represents lightness (L*), the x, y coordinates indicate the hue angle (h) and the distance from the origin to the x, y coordinates is a measure of the relative colorfulness, defined as chroma (C*). The x-axis (the a* coordinate in CIELAB) codes for the relative Green or Magenta saturation and the y-axis (the b* coordinate in CIELAB) codes for the relative Blue or Yellow saturation. The relative Magenta, Blue, Green and Yellow saturation defined along the x, and y axes in the CIELAB perceptual color space represent the polar hues that are used in MODEL 2. The origin represents middle grey with no hue value and a lightness value of 50.

**
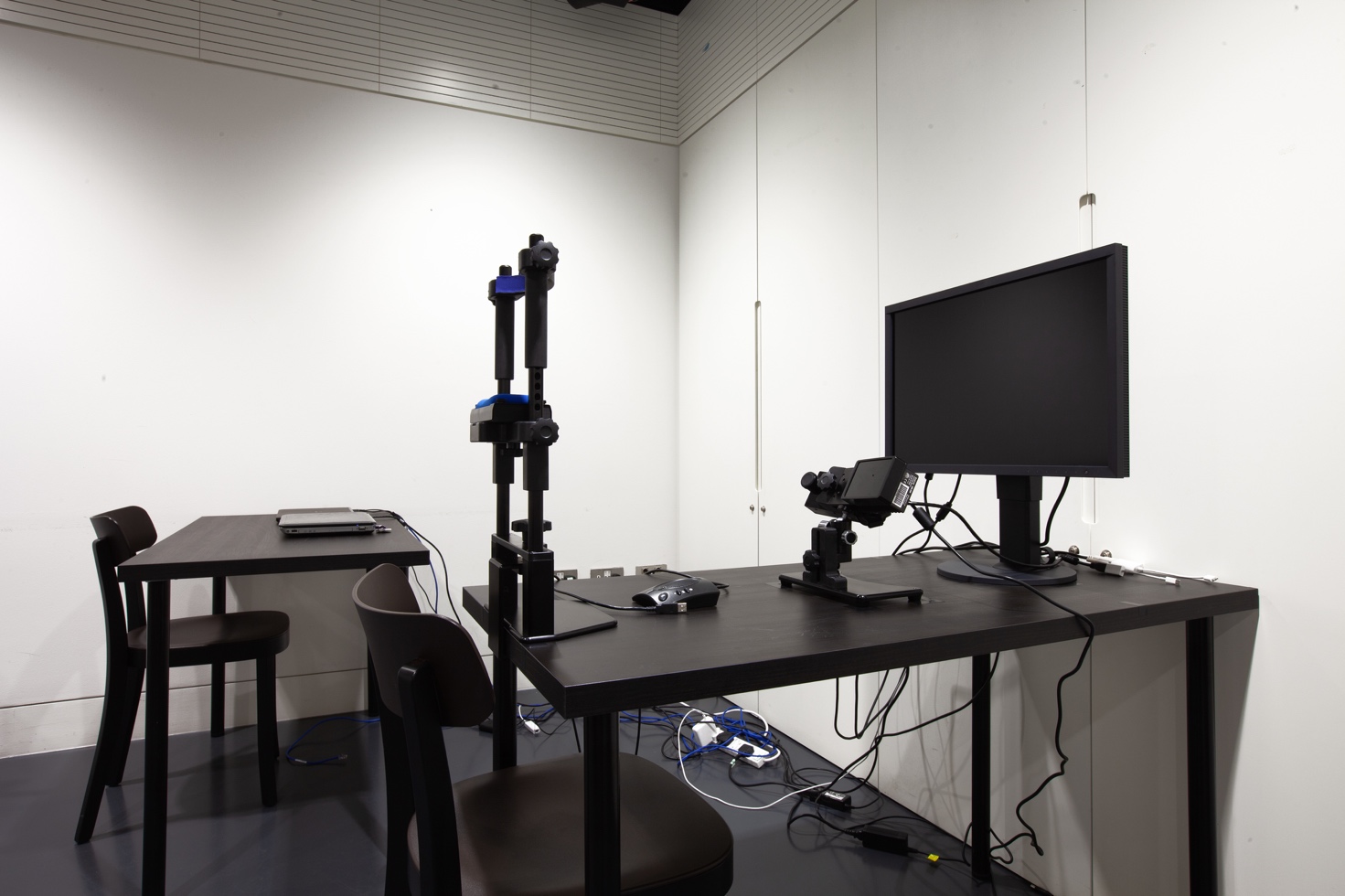
**

**Supplementary Figure 3** Set-up of the color pupillometry experiment. During the experiment the ambient light was switched off and participants were given time to adjust to the darkness of the room before the start of the first trial.


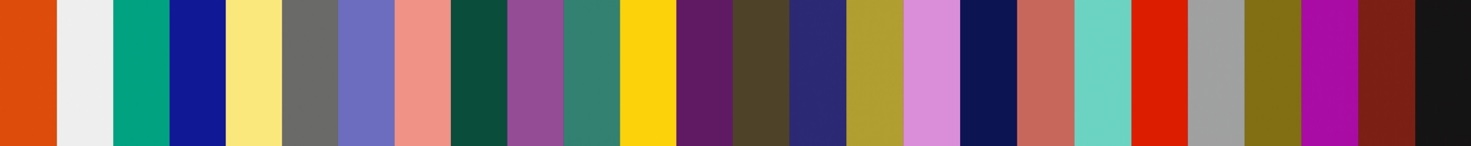


**Supplementary Figure 4** Experimental color selection, ranked in order of trial presentation from left to right. From left to right: Saturated Orange, White, Saturated Green, Saturated Blue, Light Yellow, Dark Grey, Light Blue, Light Red, Dark Green, Muted Purple, Muted Green, Saturated Yellow, Dark Purple, Dark Brown, Muted Blue, Muted Yellow, Light Purple, Dark Blue, Muted Red, Light Green, Saturated Red, Light Grey, Dark Yellow, Saturated Purple, Dark Red, Black.


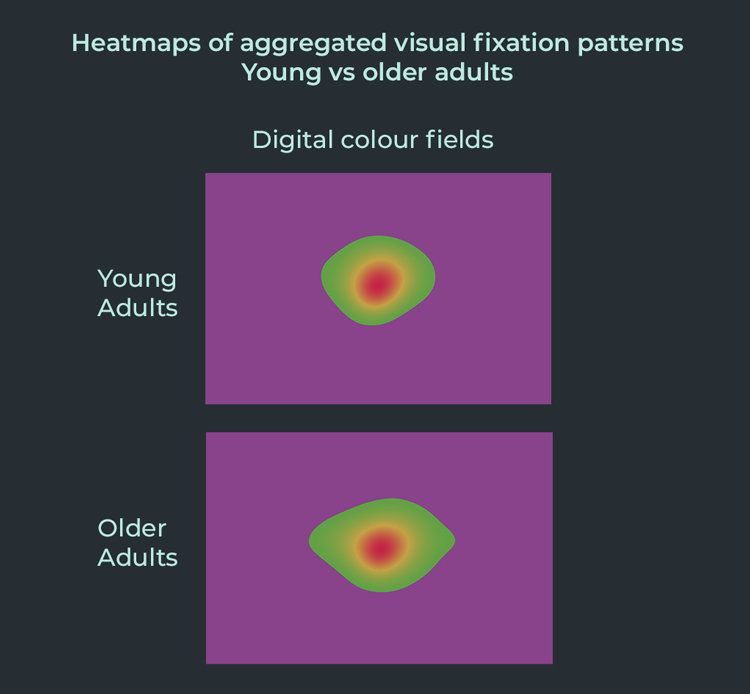


**Supplementary Figure 5** Heatmaps of aggregated visual fixation patterns across the 26 digital color fields in young and older adults. The transparent overlay colors indicate the fixation duration density, from low (green) to moderate (yellow) and high (red).
